# Reconstruction of the Recurrent Laryngeal Nerve Using an Artificial Nerve Conduit for Thyroid Cancer: A Case Report

**DOI:** 10.7759/cureus.80487

**Published:** 2025-03-12

**Authors:** Keiji Kuroki, Takuya Masunaga, Fumihide Rikimaru, Takayuki Sueta, Toshifumi Sakata

**Affiliations:** 1 Head and Neck Surgery, Fukuoka University Hospital, Fukuoka, JPN; 2 Otolaryngology, Fukuoka University Hospital, Fukuoka, JPN

**Keywords:** artificial nerve conduit, nerve reconstruction, recurrent nerve, thyroid papilloma cancer, voice function

## Abstract

The patient was a woman in her 40s who was aware of her hoarseness and was referred to our hospital. Right vocal cord paralysis was observed, and a diagnosis of papillary thyroid cancer (cT4aN1bM0) was made based on imaging tests. Total thyroidectomy (TT) and right neck dissection (ND) were performed. The right recurrent nerve, which was invaded by the tumor, was also resected, and nerve reconstruction was performed using an artificial nerve conduit (ANC). ANC can be used for nerve reconstruction in the same way as conventional autologous tissue, and there have been no reports of postoperative deterioration of language function. We have not found any reports of the use of the ANC for the reconstruction of the recurrent laryngeal nerve outside Japan, and we believe that further reports, as well as long-term follow-up and accumulation of cases, are necessary in the future.

## Introduction

The recurrent laryngeal nerve, which runs on the back of the thyroid gland, can become paralyzed due to invasion or compression of the nerve by thyroid cancer as it progresses. It has been reported in Japan that about 15% of cases of thyroid cancer treatment show vocal cord paralysis before treatment [1].

Immediate reconstruction is recommended when the recurrent laryngeal nerve is resected along with the tumor [2]. Recently, nerve anastomosis using an autologous nerve graft has attracted attention, and treatment reports have been published in Japan.

On the other hand, autologous nerve grafting is problematic because of nerve damage at the site of harvesting, and it was necessary to have a large operating theater just for harvesting. Therefore, artificial nerve conduit (ANC) was developed as a material that can be used as a scaffold for nerve regeneration.

In this study, we report a case of thyroid cancer with concomitant resection of the recurrent laryngeal nerve that underwent reconstruction using an ANC and had a good postoperative course, including some literature review.

## Case presentation

The patient was a woman in her 40s with an Eastern Cooperative Oncology Group (ECOG) Performance Status (PS) of 0 and a body mass index (BMI) of 20.4 and no special remarks in her past medical history.

Present medical history

In 2022, the patient became aware of a hoarse voice. In June 2023, a local otolaryngologist noted a large thyroid gland, and in July 2023, the patient was referred to the previous doctor for a consultation. Head and neck ultrasound and computed tomography (CT) scan revealed a tumor in the right lobe of the thyroid gland and enlarged lymph nodes in the right neck, and the patient was referred to our department for further examination. Using a laryngoscope, a right vocal cord paralysis (Video 1) was observed, and the patient was diagnosed with right papillary thyroid carcinoma cT4aN1bM0 stage 1 [3]. The patient wished to undergo curative treatment, including radioactive iodine (RAI) therapy, and was admitted to our department for total thyroidectomy (TT) and right neck dissection (ND).

**Video 1:** Flexible fiberscope The right vocal cord is paralyzed. There is no atrophy or bowing of the vocal cords.

Laboratory findings

Physical Examination

The Grade, Rough, Breathy, Asthenic, and Strained (GRBAS) scale showed hoarseness at G1R0B1A1S0. Also, a 30-mm-sized, hard, poorly mobile mass was found in the right neck, and a laryngoscopy revealed the right vocal cord was fixed in the paramedian position (PP).

Blood Test Results

Free thyroxine (FT4) and thyroid-stimulating hormone (TSH) were within normal limits, but thyroglobulin (TG) and thyroglobulin antibody (TGAb) were high (Table 1).

**Table 1:** Blood test results TSH: thyroid-stimulating hormone; FT4: free thyroxine; TG: thyroglobulin; TGAb: thyroglobulin antibody

Inspection items	TSH	FT4	TG	TGAb
Results	2.25	1.34	5.21	985
Lower limit	0.61	0.9	-	-
Upper limit	4.23	1.7	35.1	19.3
Unit	mIU/L	ng/dL	ng/mL	IU/mL

Imaging Findings

**Figure 1:**
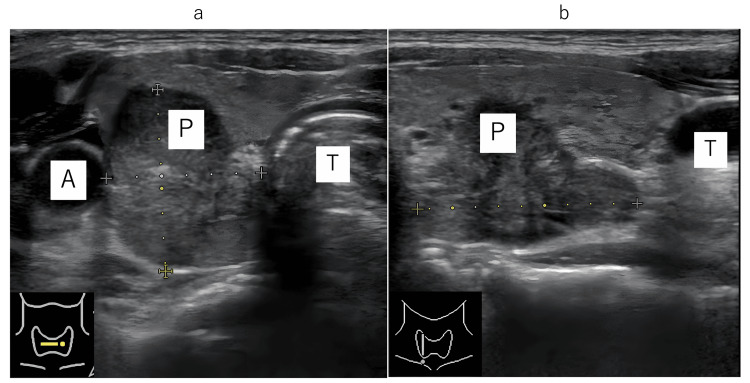
Head and neck ultrasound: (a) axial and (b) sagittal A: carotid artery; P: primary tumor; T: trachea

Ultrasound: A tumor with internal heterogeneity (Figure 1a, 1b) was observed in the middle to lower pole of the right lobe of the thyroid gland, with a maximum diameter of 26 mm in the neck.

**Figure 2:**
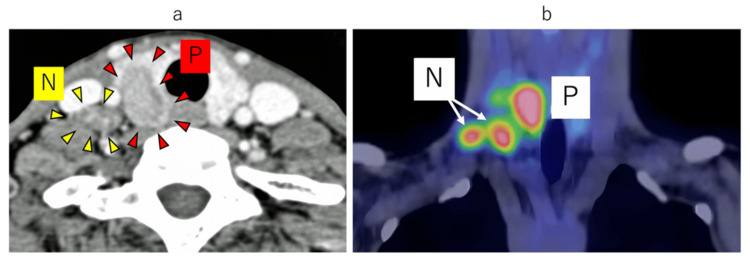
Contrast-enhanced CT axial (a) and PET-CT coronal (b) N: lymph node; P: primary tumor; CT: computed tomography; PET: positron emission tomography

CT: A primary tumor was found in the lower pole of the right lobe of the thyroid gland, which extended 23 mm in the dorsal direction from the thyroid gland (Figure 2a), and a lymph node with a short diameter of 12 mm was found in the lower neck (right level IV) behind the internal jugular vein, with an enhancement effect. A similar lymph node with a short diameter of 11 mm was also found above the clavicle (right level V).

Positron emission tomography (PET)-CT: Fluorodeoxyglucose (FDG) abnormal accumulation was observed in a primary tumor in the lower right lobe of the thyroid gland (e/dSUVmax=8.78/9.39), the enlarged lymph node on the midline of the right lower neck (SUVmax=5.17), and the enlarged lymph node above the right collarbone (SUVmax=5.08) (Figure 2b).

Treatment progress

Surgical Findings

TT and right ND (levels II-VI) were performed.

Starting from the ND, the sternocleidomastoid muscle, internal jugular vein, and accessory nerve were preserved, and the upper end of the dissection was at the level of the digastric muscle, the lower end of the dissection was at the level of the supraclavicular fossa, and the posterior end of the dissection was at the posterior border of the trapezius muscle. The metastatic lymph nodes were clustered on the posterior surface of the internal jugular vein from the right jugular vein angle and were dissected as a single mass.

**Figure 3:**
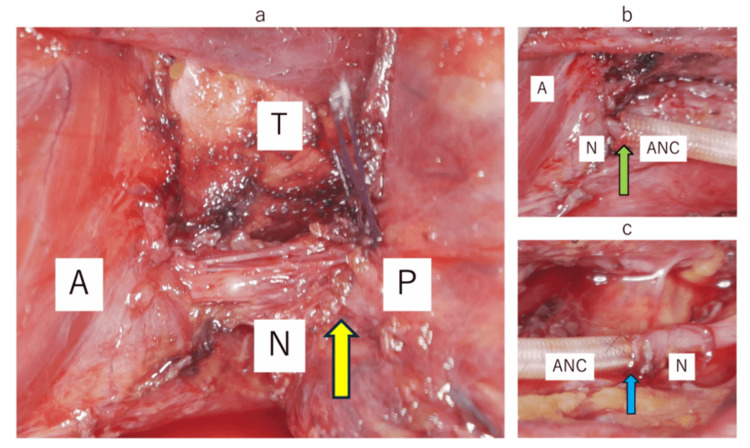
Operative findings A: anterior laryngeal muscle; N: recurrent laryngeal nerve; P: primary tumor; T: trachea; ANC: artificial nerve conduit Yellow ↑: encase; green ↑: peripheral suture; blue ↑: central suture

The thyroid cancer was mainly located on the posterior surface of the right lower pole of the thyroid gland, encasing the right recurrent nerve in a full circle (Figure 3a), and was found to be adherent to the esophageal wall. The right recurrent nerve was cut at the tumor invasion site, and TT and central ND were performed. Although part of the esophageal adventitia was also resected, there was no esophageal hiatus, and the left recurrent nerve was preserved.

The right recurrent nerve was additionally resected with a safety margin of 5 mm on the peripheral side and 2 mm on the central side. After confirming that there were no malignant findings in the pathological examination, the resection margin was sutured with a 9-0 nylon thread, with the inner diameter of the tube (Renerave®︎, Nipro, Osaka, Japan; inner diameter: 2.5 mm, length: 35 mm) penetrating the lumen of the tube by 2-3 mm under a microscope (Figure 3b, 3c). After the tube was removed, the left vocal cord paralysis and laryngeal edema were confirmed using a laryngoscope. The final pathology examination revealed right recurrent nerve invasion and multiple cervical lymph node metastases, and the patient was diagnosed with right papillary thyroid carcinoma, pT4aN1b, pEx2.

Postoperative course

**Figure 4:**
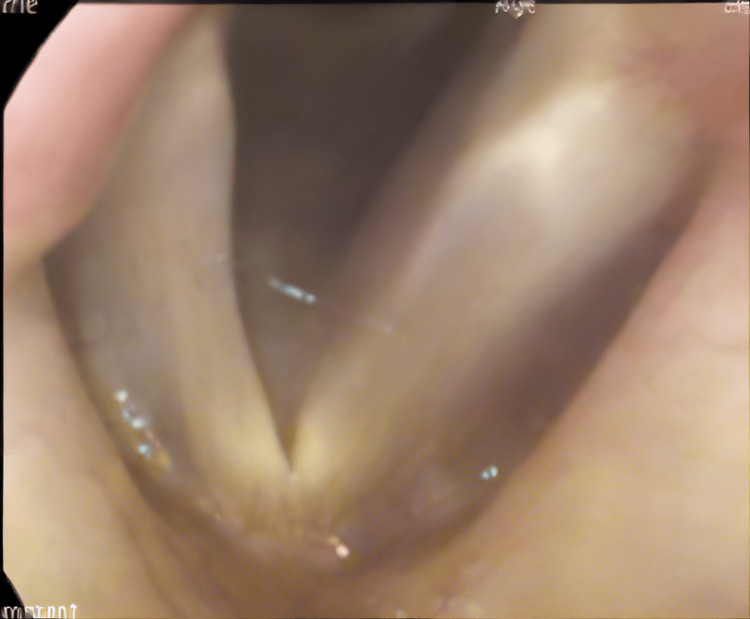
Flexible fiberscope

The patient was discharged after a period of recovery without any neck infection. One year and three months after the operation, the patient was found to have G1R0B1A1S0, with the right vocal cords fixed by PP, and no vocal cord atrophy or bowing (Figure 4), with a maximum phonation time of 23 seconds, a voice handicap index of 41 points (grand total), and a voice-related quality of life of 67.5 points (grand total), and was able to eat a normal diet.

The patient was diagnosed as being at high risk on pEx2 and underwent RAI therapy (131I-NaI 4.32GBq) at another hospital. Iodine scintigraphy showed accumulation in the thyroid bed, and a second iodine treatment is scheduled. Her condition is being evaluated through blood tests and imaging tests, and the patient is currently showing no signs of clear recurrence or worsening.

## Discussion

The incidence of thyroid cancer has been increasing rapidly in almost all developed countries since 2000. In particular, the incidence of papillary thyroid cancer has been increasing worldwide, and it is reported that it accounts for 90.4% of thyroid cancer in Japan [4]. On the other hand, the mortality rate for thyroid cancer in many countries is less than one in 100,000 people [5], and even if distant metastasis is observed in papillary thyroid cancer in patients aged 55 years or younger, it is diagnosed as stage II [3], so preserving voice function with consideration for long-term quality of life after cancer treatment is desirable. 

In our department, we select the NIM TriVantage EMG (Medtronic, Tokyo, Japan) endotracheal tube as the intubation tube during general anesthesia in order to preserve the recurrent laryngeal nerve. The usefulness of using this tube for intraoperative nerve monitoring has been reported [6], but we use it as an auxiliary means for identifying the recurrent nerve. Basically, we carefully separate the connective tissue of the tracheoesophageal sulcus, identify the cord-like structure that seems to be the recurrent nerve, and trace it to its inflow into the anterior laryngeal muscle to identify it as the recurrent nerve. In addition, as shaving is recommended [7] for tumors that remain on the surface of the nerve to ensure that there is no residual tumor, we also believe that the recurrent laryngeal nerve is protected from cancer invasion by a hard nerve sheath, and when it is possible to perform sharp dissection even if there is vocal cord paralysis, we preserve the recurrent laryngeal nerve by performing sharp dissection from the tumor. In this case, the recurrent nerve was identified in the same way, but because sharp dissection was not possible, it was resected along with the tumor. Surgical procedures for postoperative vocal cord paralysis include fat injections into the vocal cords and the use of artificial devices to move the vocal cords to the center, but these procedures take too long to perform at the same time as thyroid surgery, and there is a possibility that they will interfere with the response to complications such as bleeding during thyroid surgery. In addition, there are surgical methods that do not have a semi-permanent effect, and even if the surgery is performed in two stages, the physical burden of having to undergo general anesthesia twice and the cost of hospitalization can be a significant loss for the patient. Therefore, our department proposes immediate nerve reconstruction when performing a combined neurosurgical resection.

By immediately reconstructing the severed recurrent nerve with autologous tissue, in many cases, recovery of voice function can be expected within one year, and the arched changes and atrophy of the vocal cords are prevented, preserving voice function [8-10]. On the other hand, nerve reconstruction using ANC is useful for short gaps [11-13], and although it is an animal experiment, it is said to be superior to autologous reconstruction [14], and in facial nerve reconstruction, it shows motor function recovery comparable to autologous transplantation [15]. It is also important to suture and fix the nerve inside the ANC with a few millimeters of the nerve inserted to promote stronger attachment and wider nerve regeneration. As with reconstructing autologous tissue, it is also important not to let it dry out during surgery and to be gentle when cleaning the wound.

ANC became covered by insurance in Japan in 2013, and Nishiyama et al. [16] reported good postoperative sensory findings when it was used during combined resection of the glossopharyngeal nerve, while Nishida et al. [17] reported good postoperative voice function when it was used during combined resection of the recurrent laryngeal nerve in pediatric advanced thyroid cancer.

Since an ND was also being performed, we also considered nerve reconstruction using the ansa cervicalis, which could be secured in the surgical field, but we judged that the risk of postoperative infection with the artificial ANC was not high (BMI >20, no diabetes, no history of radiotherapy, no history of chemotherapy, no clindamycin administration, no blood transfusion, albumin >3.5) [18], and we selected the ANC as the reconstruction tissue. In addition, the reason for autologous nerve grafting is that nerve damage at the site of collection is a problem.

ANC is a medical device made of a tubular structure made of polyglycolic acid and collagen, and it serves as a scaffold for nerve regeneration in cases of nerve rupture or damage. There have been reports [19] in Japan of the use of this tube in reconstructive surgery for multiple cases of recurrent nerve combined resection, with good preservation of speech function, and a clinical trial (jRCTs:052210092) is also being conducted to verify the usefulness and safety of ANC in nerve reconstruction after cutting and combined resection of cranial nerves during resection of locally advanced head and neck cancer. Furthermore, it is also expected to be effective for gaps longer than 30 mm [20]. ANC is considered to be indicated for cases where a lateral ND is not performed and for cases where the risk of postoperative infection is not high.

## Conclusions

In this case, we used ANC as a reconstruction material in a thyroid cancer case with a confirmed recurrent laryngeal nerve palsy and were able to preserve the patient's voice function. There was no sign of wound infection after the surgery, and no recurrence of cancer was observed. There are very few cases of using ANC for recurrent nerve reconstruction around the world, and I think that long-term follow-up will be necessary in the future to see if there is any vocal fold atrophy or bowing due to delayed nerve atrophy or conduit deterioration.

There are also many issues related to ANC, such as the lack of consistency in functional recovery, the lack of comparative data with autologous transplants, and the limitations of long-term clinical verification, and we believe that further accumulation of cases and comparative studies is necessary.
